# Echocardiographic Assessment of Atrial Function: From Basic Mechanics to Specific Cardiac Diseases

**DOI:** 10.3390/jcdd9030068

**Published:** 2022-02-27

**Authors:** Katsuji Inoue, Hiroshi Kawakami, Yusuke Akazawa, Haruhiko Higashi, Takashi Higaki, Osamu Yamaguchi

**Affiliations:** 1 Department of Cardiology, Pulmonology, Hypertension & Nephrology, Ehime University Graduate School of Medicine, Toon 791-0295, Ehime, Japan; kawaka@m.ehime-u.ac.jp (H.K.); z3p1002@gmail.com (Y.A.); hingassy@yahoo.com (H.H.); yamaguti@m.ehime-u.ac.jp (O.Y.); 2 Department of Regional Pediatrics and Perinatology, Ehime University Graduate School of Medicine, Toon 791-029, Ehime, Japan; higaki@m.ehime-u.ac.jp; 3 Department of Pediatrics, Ehime University Graduate School of Medicine, Toon 791-0295, Ehime, Japan

**Keywords:** left atrial function, heart failure, pressure–volume loop, left atrial strain, atrial fibrillation, cardiac amyloidosis, adult congenital heart disease

## Abstract

The left and right atria serve as buffer chambers to control the flow of venous blood for ventricular filling. If an atrium is absent, blood does not flow effectively into the ventricle, leading to venous blood retention and low cardiac output. The importance of atrial function has become increasingly recognized, because left atrial (LA) function contributes to cardiac performance, and loss of LA function is associated with heart failure. LA volume change has been used for LA function assessment in experimental and clinical studies. In conjunction with LA pressure, the LA pressure–volume relationship provides a better understanding of LA mechanics. LA strain measurement by speckle tracking echocardiography was introduced to evaluate three components of LA function as a (booster) pump, reservoir and conduit. Furthermore, increasing evidence supports the theory that LA reservoir strain has prognostic utility in various cardiac diseases. In this review, we summarize LA contribution to maintain cardiac performance by evaluating LA function with echocardiography according to our experiences and previous reports. Furthermore, we discuss LA dysfunction in challenging cardiac diseases of cardiac amyloidosis and adult congenital heart disease.

## 1. Introduction

The atria work as buffer chambers to receive blood from the venous system in a controlled manner and to effectively deliver it to the ventricles. If an atrium is absent, the ventricle carries a considerable hemodynamic burden to maintain cardiac performance. Under such circumstances, preservation of cardiac output depends only on ventricular systolic and diastolic function; otherwise, forward flow is unavoidably produced by an increased pressure in the upper venous system. For example, when the pressure in the pulmonary vein is elevated to force a forward flow into a failing left ventricle, patients complain of heart failure symptoms, such as shortness of breath and dyspnea on exertion. The scenario demonstrates the importance of the left atrial (LA) contribution to overall cardiac function.

The left atrium contributes to cardiac performance through three components, functioning as a (booster) pump, reservoir and conduit. LA pressure and volume change instantaneously during the cardiac cycle. The resultant LA pressure–volume curve shows a figure-eight configuration, which provides important information regarding LA active and passive function.

Echocardiography was first used to assess LA size and function in clinical practice [1]. Two-dimensional (2D) echocardiography can be used to quantify LA size, and the LA volume index estimated by 2D echocardiography is a well-known diagnostic and prognostic parameter in patients with heart failure [2,3]. In terms of LA functional assessment, historically, Doppler echocardiography, particularly the pulmonary vein signal, is utilized to understand LA pressure and function. As a screening tool, pulmonary vein flow is relatively underused compared with transmitral flow to evaluate LA function; however, the waveform of pulmonary vein flow enables assessment of the LA pump, reservoir and conduit functions [4,5].

The importance of the atrial contribution to cardiac function has become increasingly recognized in clinical practice. In this review, we summarize LA contribution to prevent heart failure by evaluating LA function with echocardiography according to our experiences and previous reports. Furthermore, we discuss LA dysfunction in challenging cardiac disorders of cardiac amyloidosis and adult congenital heart disease (ACHD).

## 2. Basic Mechanics of the Left Atrium

The pressure–volume relationship is the gold standard for evaluating myocardial work. Suga presented the concept of the pressure–volume loop area as a novel marker of myocardial work in the left ventricle [6]. Russel, Smiseth, and colleagues developed a noninvasive method to quantify left ventricular (LV) work from the pressure–strain loop area using speckle tracking echocardiography [7].

**Figure 1:**
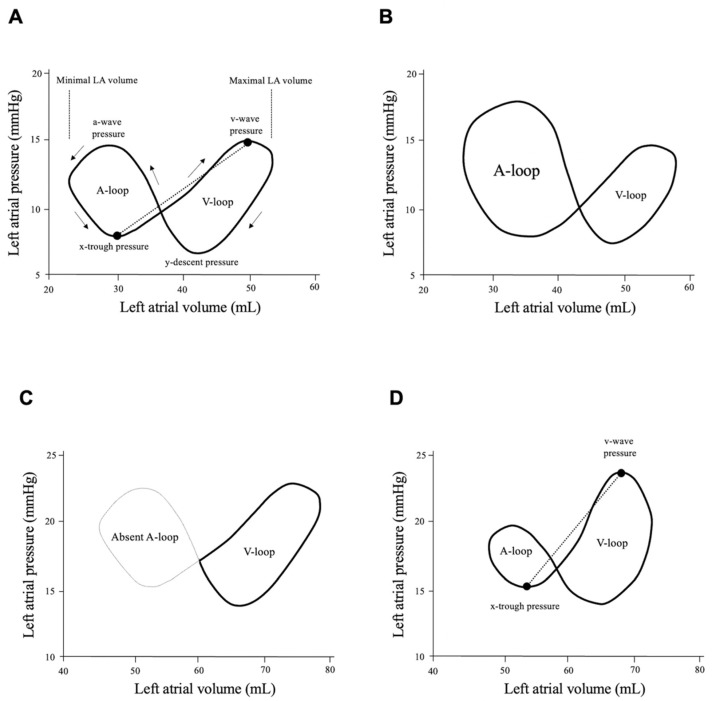
Left atrial pressure–volume loop curve. (**A**) Normal left atrial pressure–volume loop consisting of A- and V-loops. The A-loop is produced by LA contraction (booster pump function) and relaxation (early reservoir function), while the V-loop is produced by LA dilatation (late reservoir function) and emptying (conduit function). LA pressure includes the x-trough and v-wave pressures, and the slope connecting the two pressure points (dotted line) on the loop is defined as the LA stiffness index. (**B**) During afterload increase. When systolic blood pressure increases from 120 to 180 mmHg for instance, left ventricular (LV) systolic and diastolic function worsen due to afterload increase. The A-loop area immediately enlarges to pressure-overloaded LV dysfunction, resulting in the maintenance of stroke volume without a critical elevation in LA pressure. (**C**) Atrial fibrillation occurrence. Atrial fibrillation promotes atrial myopathy, resulting in LA dilatation, functional impairment and perhaps elevated mean LA pressure. The A-loop disappears during atrial fibrillation, and it mainly depends on ventricular function to maintain cardiac performance. (**D**) Left atrial stiffening. The pressure–volume loop in a case of cardiac amyloidosis. Abnormal amyloid proteins deposit into the myocardial wall, including the left atrium. LA, left atrial; LV, left ventricular.

Regarding the LA pressure–volume relationship, the loop typically has a figure-eight pattern (Figure 1A), with the two loops consisting of the A- and V-loops [8]. The A-loop rotates counterclockwise, which corresponds to LA active contraction and relaxation, while the V-loop rotates clockwise, which corresponds to LA passive dilatation and emptying.

The A-loop area represents LA active function to maintain stroke volume and normal LA pressure despite afterload increase [9]. Although an increase in afterload limits LV work, augmentations of LA pump function (booster function) and LA active relaxation (early reservoir function) immediately react as compensatory mechanisms to prevent ‘afterload mismatch’ (Figure 1B). However, if atrial fibrillation (AF) is already present, heart failure could develop, because the A-loop is absent due to atrial myopathy (Figure 1C) [8].

The V-loop represents LA passive function. The first half of the upsloping V-loop occurs during the LV ejection period, and the slope begins at the time point of x-trough pressure and ends at that of v-wave pressure. During the LV ejection period, LV myocardial fiber shortens longitudinally, and in turn, the LA wall lengthens as a source of the LA late reservoir function.

The second half of the downsloping V-loop begins at mitral valve opening. After the mitral valve opens, the left atrium behaves as a conduit by producing forward blood flow through LA elastic recoil as well as LV diastolic function. The left atrium reflects LV systolic and diastolic function because of the anatomical continuity between the two chambers [10].

When we focus on LA reservoir function, the left atrium works as a reservoir to maintain cardiac output. An experimental study elegantly revealed that LA reservoir function was determined by LA relaxation, LV longitudinal shortening and LA stiffness [11]. In this phase, the LA pressure rises from the x-trough to v-wave pressure. Importantly, the ratio of the pressure gradient from the x-trough to v-wave pressure to LA volume increase is a surrogate of LA chamber stiffness (Figure 1D). The concept of the LA pressure–volume relationship provides a better understanding of LA intrinsic function as well as LA passive function supported mainly by adjacent LV work.

In this case, the slope connecting the x-trough pressure to the v-wave pressure on the loop becomes steeper than that in other cases with a compliant left atrium (Figure 1A).

## 3. Atrial Function in Cardiovascular Diseases

It is recognized that LA function is important to prevent heart failure, particularly if LV function deteriorates. In cases with LV dysfunction, LA dilatation during ventricular systole, which is one of the components of LA ‘reservoir function’, is limited due to reduced LV longitudinal systolic shortening. Suga reported that LA reservoir function was an important determinant of cardiac output [12]. Thus, heart failure could develop unless a compensatory mechanism operates. Under the circumstances, the left atrium immediately responds by operating its intrinsic ‘pump function’ to expand a diseased left ventricle and maintain normal cardiac output via the Frank–Starling mechanism [13]. The augmented LA pump function is also linked with enhanced LA relaxation, thus effectively pulling blood from the pulmonary vein to the left atrium [14]. The occurrence of AF is directly associated with an increased incidence of heart failure, especially in patients with LV dysfunction [15,16]. Ablation of the AF is effective to obtain LA reverse remodeling and is expected to reduce the occurrence of heart failure.

LA dilatation is a marker of chronicity of LV diastolic dysfunction. An LA volume index (LAVi) >34 mL/m^2^ is an important parameter in estimating LV filling pressure [2]. Meanwhile, AF itself promotes LA dilatation irrespective of LV filling pressure. LA enlargement coexists with LA functional impairment.

**Figure 2:**
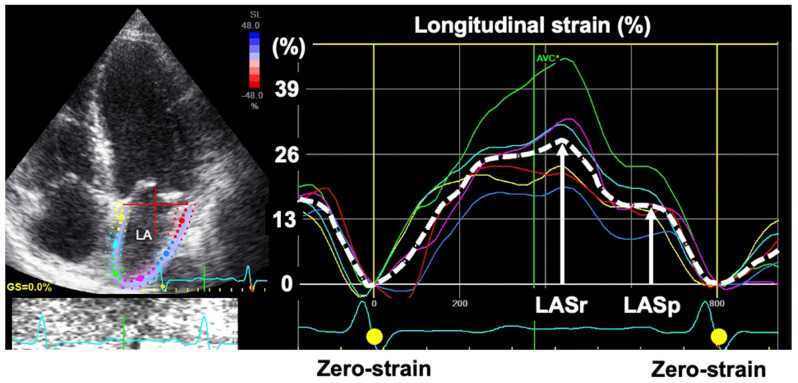
Calculation of left atrial strain. LA strain can be analyzed from a non-foreshortened apical four-chamber view. The zero-strain reference is set at end-diastole according to the recommendation by Badano et al. [17], enabling one to obtain LA strain value even in patients with atrial fibrillation. LA reservoir strain (LASr) is calculated as difference between end-diastole and onset of early LV filling, while LA pump strain (LASp) as difference between onset of atrial contraction and onset of late LV filling. LA, left atrial, LV, left ventricular.

LA strain by speckle tracking echocardiography (STE) can be used to evaluate LA function. The EACVI/ASE/Industry Task Force provides standardization of LA strain measurement for clinical and scientific purposes [17]. For the measurement, the Task Force recommends that the zero-strain reference for LA strain curves should be end-diastole, as shown Figure 2. The LA strain curve has two positive peaks corresponding to LA reservoir and pump functions.

LA functional assessment is emerging to diagnose and predict heart failure in patients with underlying heart diseases. We recently collaborated to conduct a multicenter study of 322 patients with cardiovascular disease of different etiologies [18], resulting that LA reservoir strain < 18% and LA pump strain < 8% predicted elevated LV filling pressure better (*p* < 0.05) than LA volume [18]. Furthermore, LA pump strain > 14% identified normal LV filling pressure with 92% accuracy in patients with preserved LV ejection fraction. These results suggest that LA reservoir strain is an alternative marker to LA volume index for identifying elevated LV filling pressure. In addition, a preservation of LA pump strain indicates LA compensation to maintain normal LV filling pressure in patients with underlying cardiac diseases.

As LA remodeling progresses, LA stiffness increases. The assessment of LA stiffness has been studied from the LA pressure–volume relationship, as mentioned above. Reddy Y et al. reported that LA stiffness progressively increased with LV diastolic dysfunction and AF burden, leading to poor outcomes in patients with heart failure with preserved LV ejection fraction [19]. Cardiac amyloidosis is also a representative cardiac disease with stiff LA syndrome. When we visually focus on LA behavior by echocardiography, LA dilatation is somewhat restricted at the LA reservoir phase in patients with cardiac amyloidosis. This is due to amyloid infiltration in the LA wall leading to progressive loss of atrial function and increased stiffness [20].

In contrast to the left atrium, right atrial (RA) structure and function are not routinely evaluated with echocardiography in clinical practice. In adult congenital heart disease (ACHD), over time, the right-sided heart is often more diseased rather than the left-sided heart. Patients with ACHD suffer from right-sided heart failure presenting as anasarca, ascites and general fatigue. Right ventricular (RV) dilatation is usually observed, and echocardiography enables the estimation of RV size as well as function. The right atrium is also dilated concomitantly with RV remodeling, although the assessment of RA structure and function is less studied in patients with ACHD. Furthermore, the prevalence of AF is rising precipitously with the increase in the aging population, and the right atrium is progressively remodeled. The success rate of AF ablation is unsatisfactory in patients with ACHD with complex cardiac structure and previous surgery.

## 4. Left Atrial Structure and Function in Atrial Fibrillation

Atrial fibrillation is the most common arrhythmia, and the number of patients with AF has been increasing gradually and steadily worldwide [21,22]. The increasing incidence of AF has contributed to rising health care costs because of the association of AF with stroke, heart failure, and overall mortality [21,22,23]. Therefore, better preventive and therapeutic steps for the management of this arrhythmia are needed. AF initiates self-perpetuating changes in the structural, functional, and electrophysiological properties of the atrium and promotes atrial remodeling (AF begets AF) [24]. LA structural and functional cardiac abnormalities, including chamber dilatation and systolic/diastolic dysfunction, are all potential risk factors for AF [25,26,27,28,29]. Furthermore, LA structural and electrophysiological remodeling are key markers for risk stratification in patients with AF [1,26,27,28,30,31,32,33,34].

Considerable evidence exists regarding the usefulness of echocardiographic LA structural and functional parameters for risk stratification (stroke, heart failure, and overall mortality) in patients with AF. In particular, LAVi is a well-established parameter for risk stratification of cardiovascular events and recurrence of catheter ablation (CA) for AF. Osranek et al. reported the results of very long-term follow-up (median 27 years) of 46 patients with well-documented, clinically defined, lone AF [32]. They revealed that patients with large LA volume (LAVi ≥ 32 mL/m^2^) at baseline had a significantly worse event-free survival after adjustment for age and clinical risk factors (adjusted HR, 4.46). In a systematic review and meta-analysis, Njoku et al. assessed whether LA volume was an independent predictor of AF recurrence following CA, and found that patients with AF recurrence following CA had a higher mean LA volume/LAVi compared with patients with no recurrence [31]. In contrast, many studies have revealed a reduction in LA enlargement after restoration of sinus rhythm by CA (reverse remodeling), and LA reverse remodeling was thought to be a marker for better outcomes (lower recurrence rate) of CA for AF [33,35,36,37,38,39]. In our recent work, we investigated whether the degree of LA volumetric reverse remodeling was associated with long-term outcomes (AF-free survival) after initial CA for AF in 140 patients with LA enlargement (LAVi ≥ 34 mL/m^2^) at baseline [33]. We found that more than half of the patients (54%) had a normal LA volume (LAVi < 34 mL/m^2^) at 1 year follow-up after CA, and those patients had better long-term outcomes (lower recurrence rate) after CA than patients who did not have a normal LA volume. Recent guidelines report the normal ranges and severity partition cutoffs of LA volume abnormality assessed by echocardiography [40]; an LAVi of 34 mL/m^2^ can be used as a clear cut-off for risk stratification in a variety of clinical settings in patients with AF.

**Figure 3:**
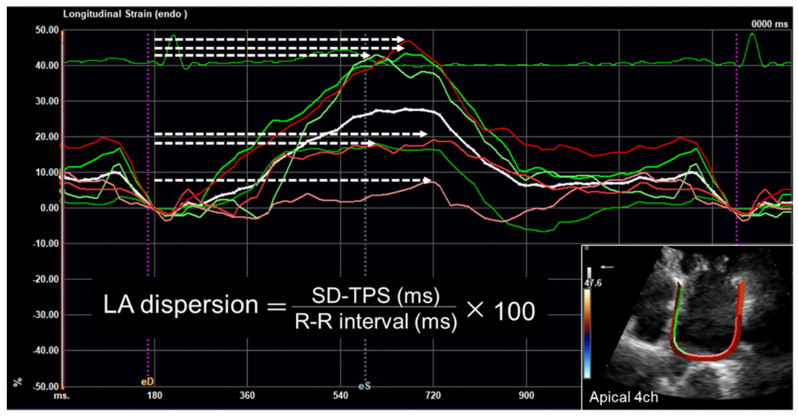
Calculation of left atrial dispersion. LA dispersion is defined as the standard deviation of the time to peak positive strain corrected by the R-R interval (%). White arrows indicate contraction durations defined as the time from the R wave on the electrocardiogram to the maximal time of positive deformation in each LA segment. LA, left atrial.

LA strain assessed by STE can detect early impairment of LA function, such as reduced LA reservoir and (booster) pump function [41,42]. Kuppahally et al. carried out a validation study on the relationship between LA strain and cardiac magnetic resonance and revealed that LA strain was inversely associated with LA fibrosis [43]. Moreover, STE can accurately assess regional myocardial function; thus, LA strain assessed by STE may also be used to evaluate disturbances in the timing of LA contraction (as a LA mechanical dispersion) (Figure 3), which reflect the presence of atrial fibrosis and electrophysiological disorders [44,45]. Watanabe et al. demonstrated that LA mechanical dispersion was increased significantly in patients with low-voltage zones evaluated by electro-anatomical mapping, and the severity of LA dispersion was related to the LA conduction delay in patients undergoing CA for AF [44]. These findings support the conclusion that STE can be a useful tool for evaluation of LA remodeling. Indeed, recent studies demonstrated that LA strain, mainly LA reservoir strain, can be a useful marker for risk stratification of AF in a variety of cardiac conditions [46,47,48,49,50,51,52,53,54,55], and can also provide incremental diagnostic and therapeutic value for AF management over LA enlargement [46,47,48,51]. Furthermore, LA dispersion is greater in patients with AF than in healthy individuals [56,57,58,59], and increases in proportion to the duration of AF [56]. LA dispersion predicts progression from paroxysmal to persistent AF [60]. In a community-based study, Kawakami et al. revealed that LA function and dispersion obtained from STE provided incremental information on LA volume and function in the prediction of new-onset AF [61]. LA strain assessed by STE has been also useful in predicting outcomes of AF treatment, including defibrillation and CA. From a functional point of view, LA reservoir strain calculated by STE is considered one of the strongest predictors of AF recurrence [53,55,62]. A recent meta-analysis proved that LA reservoir strain was strongly associated with recurrence of AF after adjusting for age, gender, AF duration, and LA volume, with an excellent predictive value (area under the receiver operating curve of 0.80) [62]. Assessment of LA dispersion using STE may also forecast recurrence after CA for AF. Sarvari et al. evaluated the association of LA dispersion with recurrence after CA for AF in patients with normal LV function and without severe LA enlargement, and demonstrated that LA dispersion was more pronounced in patients with recurrence of AF after CA compared with those without recurrence, and it predicted AF recurrence after adjustment for age, LA volume, e’, and LA function [63]. Although CA is an effective therapy for AF, CA can lead to injury to the atrial myocardium and impairments in neurohormonal functions, leading to “stiff LA syndrome” [64,65]. Therefore, evaluating LA volumetric and functional changes is important after CA for AF, especially in patients with long-standing persistent AF and heart failure. Moreover, reduced LA function and dispersed LA contraction pattern assessed by STE can also contribute to the risk stratification of stroke events in patients with AF [66,67]. Obokata et al. reported that LA strain had an incremental advantage over the CHA_2_DS_2_-VASc score for predicting the risk of stroke in patients with AF [66]. The greatest advantage of LA strain is the ability to detect early LA function and asynchrony in patients without LA enlargement [51,57,61,63].

## 5. Left Atrial Function in Cardiac Amyloidosis

Cardiac amyloidosis is characterized by extracellular amyloid infiltration in the left ventricle, resulting in a restrictive pathophysiology. Amyloid deposition in the left ventricle causes LV wall thickening and increased ventricular filling pressure, which lead to LV diastolic dysfunction. It has also been reported that amyloid is pathologically deposited not only in the left ventricle, but also in the left atrium. The left atrium in cardiac amyloidosis is hemodynamically impaired similarly to the left ventricle. Amyloid infiltration into the LA wall causes it to thicken and stiffen, increasing its size and impairing its ejection force and strain [68]. By observing and evaluating LA behavior, we can learn much about the pathophysiology of cardiac amyloidosis.

Echocardiography is a key imaging modality in diagnosing cardiac amyloidosis. LA dimension or volume is a simple parameter, which indicates a chronic elevation of LA pressure. However, LA size is insufficient to obtain detailed information about LA function. Several studies with STE demonstrated that LA systolic contraction, reservoir and conduit functions were impaired in cardiac amyloidosis [69,70]. Recently, Bandera and colleagues [20] reported substantial impairment of the three phasic functional atrial components (median reservoir strain 8.9%, conduit strain 6.5%, pump strain 4.0%) evaluated by STE. Furthermore, they demonstrated that patients with amyloidosis with sinus rhythm whose atrial contraction was absent had a poorer prognosis compared with those with sinus rhythm and effective mechanical contraction.

Since the ATTR-ACT study [71], which showed that treatment with tafamidis improved the prognosis of cardiac amyloidosis, early diagnosis and phenotyping of cardiac amyloidosis has become more important. Amyloidosis is a disease characterized by LV hypertrophy, but it is often difficult to differentiate from other diseases presenting with LV hypertrophy, such as hypertrophic cardiomyopathy, hypertensive heart disease, and athlete’s heart. In cardiac amyloidosis, the characteristic regional differences in LV longitudinal strain from cardiac base to apex are useful in differentiating the diseases. LV longitudinal strain is preserved at the apex and reduced significantly at the mid and basal segments. This strain pattern with STE is called ‘apical sparing’ and is specific to cardiac amyloidosis [72,73,74]. Regarding the left atrium in cardiac amyloidosis, Rausch et al. [75] reported that LA strain parameters at three phases were significantly reduced in the amyloidosis cohort compared to the hypertensive heart disease cohort. A reservoir strain cut-off value of 20% was 86% sensitive and 89% specific for detecting cardiac amyloidosis compared to hypertensive heart disease in their analysis.

**Figure 4:**
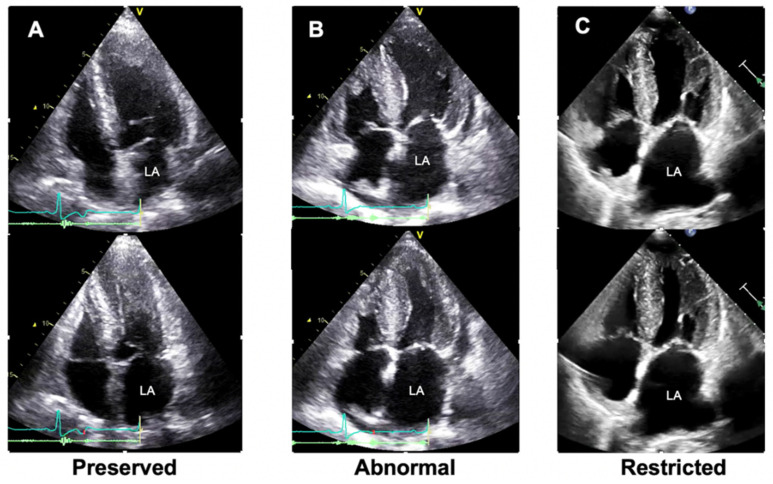
Visual assessment (grading) of left atrial reservoir function. (**A**) A case of hypertrophic cardiomyopathy. (**B**,**C**) Two cases of cardiac amyloidosis. The upper panel in each case is a frame when the LA size is minimized, while the lower panel is a frame when the LA size is maximized. In a patient with hypertrophic cardiomyopathy, the LA dilatation grade (Inoue grade) was preserved (**A**). In contrast, two patients with cardiac amyloidosis showed abnormal (**B**) and restricted (**C**) LA dilatation, indicating LA stiffening. LA, left atrial.

Recently, we demonstrated the usefulness of visual assessment of the left atrium to differentiate cardiac amyloidosis from hypertrophic cardiomyopathy [76]. As shown in Figure 4, we classified the LA dilatation motion into three grades (preserved: grade 1, abnormal: grade 2, and restricted: grade 3) based on the apical four-chamber view and defined it as the LA dilatation grade (Inoue grade). Among 127 subjects, all 57 (45%) who presented with Inoue grade 1 had hypertrophic cardiomyopathy. In contrast, 28 patients (22%) were classified as Inoue grade 3, 20 of whom had cardiac amyloidosis. Thus, patients with cardiac amyloidosis had a higher Inoue grade than those with hypertrophic cardiomyopathy (*p* < 0.01). This diagnostic parameter is not only an indicator for the diagnosis of cardiac amyloidosis, but also a predictor of prognosis.

Impaired LA function in cardiac amyloidosis leads to decreased cardiac output and the development of heart failure. Another problem related to the clinical course of cardiac amyloidosis is the high incidence of arrhythmias. In particular, AF is commonly encountered and is the most troublesome arrhythmia in this condition. Donnellan et al. reported that AF occurred in 265 (69%) of 383 patients with amyloidosis in a retrospective cohort study [77]. They also identified risk factors associated with the development of AF, including older age, advanced cardiac amyloidosis stage, and higher LA volume index. AF can also cause cardiogenic stroke due to thrombus formation in the left atrium (mainly in the LA appendage). In general, a thrombus is unlikely to form in the left atrium when sinus rhythm is maintained, but in cardiac amyloidosis, thrombus formation can occur even in sinus rhythm. Martinez-Naharro et al. [78] reported that the prevalence of intracardiac thrombi was 6.2% in 324 patients with cardiac amyloidosis (256 males, 79%; 71 ± 11 years of age). Of the 20 patients with intracardiac thrombi, 13 had AF, and seven had sinus rhythm. All patients with AF and intracardiac thrombi were receiving long-term anticoagulation therapy. Therefore, cardiac amyloidosis is a disease that requires careful attention to thromboembolic events, even in patients with normal sinus rhythm or receiving anticoagulation therapy.

Echocardiography has the potential to enable early diagnosis of cardiac amyloidosis. By focusing not only on the left ventricle, but also on the left atrium, we can better understand the cardiac pathology and provide appropriate treatment for patients with cardiac amyloidosis.

## 6. Atrial Structure and Function in Adult Congenital Heart Disease

The diagnosis and management of congenital heart disease (CHD) has been a major success story of modern medicine. More than 90% of children with CHD survive into adulthood, and the number of adults with congenital heart disease (i.e., ACHD) is increasing [79]. It is recognized that CHD is associated with lifelong comorbidity that impacts health services utilization and costs. In particular, heart failure (HF) is a serious problem affecting 20–50% of the ACHD population [80]. Diastolic dysfunction correlates with poor prognosis in various forms of ACHD [81]. In adult acquired heart disease, the assessment of LA function has recently emerged as a powerful parameter of diastolic dysfunction [82]. Although the application of these LA functional assessments to patients with ACHD remains questionable, this knowledge should be actively pursued. The application of these LA functional assessments to patients with ACHD remains questionable. We will focus on “Atrial septal defect” and “Fontan Palliation”, two common forms of ACHD, and review the current understanding of the assessment of atrial structure and function in ACHD.

### 6.1. Atrial Septal Defect

Traditionally, an atrial septal defect (ASD) has been repaired surgically. Recently, however, percutaneous transcatheter device occlusion has been used. A previous report showed that there was more significant intra-atrial systolic dyssynchrony with device closure than with surgical closure in strain imaging, suggesting that the bulky device impairs synchronous contraction [83]. In addition, at long-term follow-up, LA reservoir, conduit, and contraction functions were reduced in the device closure group compared with the healthy control group, but there was no difference in these functions in the surgical closure group. These data after ASD treatment suggest that device closure negatively affects LA function [84]. Although it has been reported that the annual incidence of new-onset AF in patients with ASD after device closure was 4.1% [85], the prognostic impact of the reduced LA function remains unknown, and further evidence must be accumulated. On the other hand, a study investigating pre-procedural risk factors for paroxysmal AF occurring after ASD device closure found that the combination of the standard deviations of RA time to peak strain, a marker of RA dyssynchrony, and the RA expansion index, a marker of RA reservoir function, provided a stronger estimate of paroxysmal AF independently of LA dysfunction [86]. These findings highlight the importance of understanding both LA and RA function in predicting the prognosis of patients with ASD.

**Figure 5:**
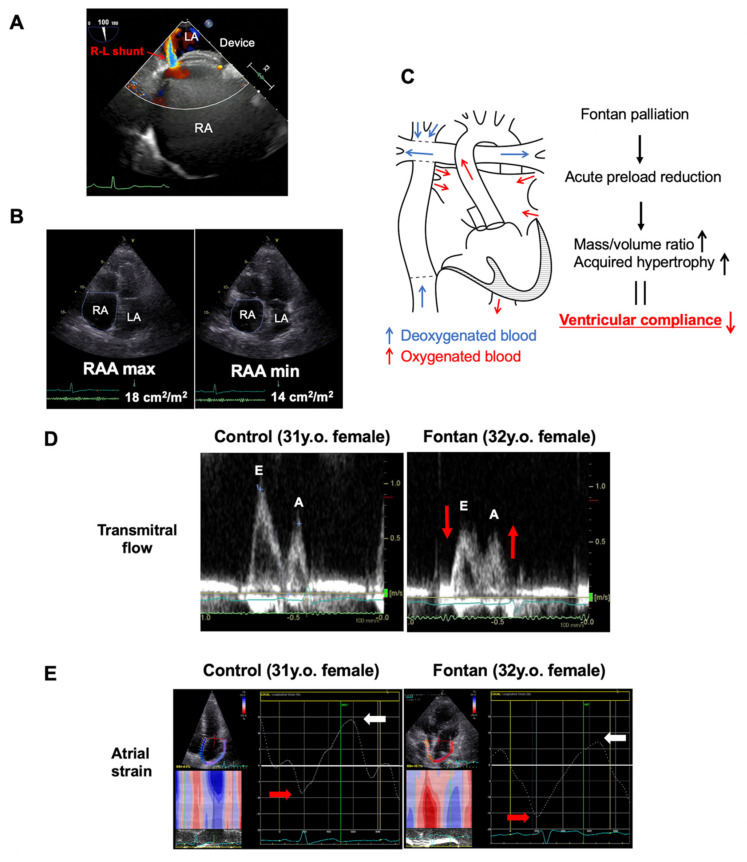
Atrial function in atrial septal defect and Fontan palliation. (**A**) TEE showing a right-to-left (R-L) shunt from a residual leak after ASD device closure with RA dysfunction. (**B**) Four-chamber views of end-systole (RAA max) and end-diastole (RAA min). The decreased RA fractional area change (FAC) suggests RA dysfunction. (**C**) Illustration of the Fontan circulation and the process of impairment of systemic ventricular relaxation after Fontan palliation. (**D**) Transmitral Doppler early diastolic (E-wave) and late diastolic (A-wave) velocities in a control subject, and the SV peak E-wave and A-wave inflow velocities in a patient with Fontan palliation (EC-TCPC). (**E**) Representative atrial strain curves in a patient with Fontan palliation (EC-TCPC) and a control subject. Peak negative (red arrow) and positive (white arrow) atrial strain are noted. TEE, transesophageal echocardiography; ASD, atrial septal defect; RA, right atrial; RAA, right atrial area; EC-TCPC, extracardiac total cavopulmonary connection.

Although rare, RA dysfunction due to impaired RV dilatation can lead to a right-to-left shunt via ASD. A case report described a patient who underwent pulmonary valvuloplasty for pulmonary stenosis with RV hypertrophy and developed a right-to-left shunt via ASD [87]. STE indicated an impaired reservoir function of the RA with a slight increase in booster function. However, STE analysis is not always possible due to difficulty in visualizing the free wall of the RA. In a case with a residual leak after ASD device implantation, RA dysfunction caused a right-to-left shunt (Figure 5A). In this case, STE analysis was difficult, but the enlarged RA area and decreased RA fractional area change suggested RA dysfunction (Figure 5B) [88].

### 6.2. Fontan Palliation

Fontan [89] reported successful performance of a right-sided cardiac bypass in patients with a functional single ventricle (SV) for the first time in 1971. Since then, with modifications in operative techniques and postoperative management, early survival has improved [90,91,92]. However, little has been investigated regarding atrial function late after the Fontan palliation.

By separating the pulmonary and systemic circulations, the Fontan palliation imposes an acute preload reduction on a previously volume-loaded ventricle. After the Fontan palliation [93,94], an increased mass/volume ratio and acquired hypertrophy of the ventricles occur, with concomitant impairment of systemic ventricular relaxation (Figure 5C). A persistent late impairment of isovolumic relaxation time and diminishing SV peak early diastolic velocity compared to controls have also been reported [95], suggesting a trend towards reduced ventricular compliance. In a previous study, the SV peak early diastolic (E-wave) was decreased, and the late diastole (A-wave) inflow velocity was increased compared with that in healthy subjects [96] (Figure 5D). This reliance on increased atrial contractility may be a response to the elevated ventricular end-diastolic pressures after the Fontan palliation. These findings were demonstrated in STE-based studies [96,97]. The SV atrial deformation parameters were significantly altered compared with healthy subjects, with markedly impaired conduit strain, reduced reservoir strain, and increased reliance on active strain for ventricular filling (Figure 5E). These differences may have important implications for their long-term outcomes; further studies are needed to clarify the role of atrial function in SV cardiac performance.

## 7. Conclusions

According to recent developments in diagnostic, therapeutic and preventive medicine, the survival rate has improved in patients with heart failure. However, the number of individuals with heart failure is increasing, because cardiac structural and functional remodeling still exists despite medical and interventional therapies. Atrial enlargement and dysfunction indicate a history of hemodynamic burden and disease severity. The assessment of atrial structure and function by echocardiography enables an understanding of the underlying risk of heart failure, thus promoting early preventive and therapeutic interventions to decrease heart failure occurrence.

## Data Availability

No datasets were analyzed in this review article.
